# The cumulative proportion of children receiving social care services in England: a whole population administrative data cohort study.

**DOI:** 10.23889/ijpds.v7i3.1895

**Published:** 2022-08-25

**Authors:** Matthew Jay, Louise Mc Grath-Lone, Rachel Pearson, Ruth Gilbert

**Affiliations:** 1 Administrative Data Research Unit – Wales, Welsh Government

## Objectives

Children who receive social care services have high levels of need, but the proportion of the population they comprise is unknown. We aimed to estimate the cumulative proportion of children in England who ever become looked after (CLA) or placed on a child in need (CiN) plan by age 18.

## Approach

We analysed longitudinal administrative children’s social care data: the CLA dataset (available from 1992) and the CiN census (from 2011/12) which respectively contain information on all children in England who become CLA and are assessed as being CiN. The number of CLA in birth cohorts from 1992 to 2016 was divided by Office for National Statistics population denominators to estimate the overall cumulative proportion and by EthPOP denominators to describe ethnic variation. Because of incomplete coverage of CiN data, we combined estimates from different birth cohorts, making reductions to the numerator to address left-censoring.

## Results

The cumulative proportion of children who became CLA before their 18th birthday was 3% for those born in 1992, rising to 4% for those born in 2000. For subsequent cohorts (who had not yet reached age 18), the cumulative proportion by earlier ages also rose over time, but plateaued with the 2012 birth cohort. The cumulative proportion varied by ethnicity: among children born in 2000, it was 18% for those of other ethnicity (many of whom were unaccompanied asylum seeking children), 11% for black children and 7% for those of mixed ethnicity. Most children first became looked after before age 2, with a secondary peak around age 16. Finally, we estimated that 42% of all children in England were recognised as CiN before age 18.

## Conclusion

Significant proportions of children receive social care services in England. The ethnic variation we observed may indicate systematic differences in how the care system responds to CLA from different backgrounds. These results have implications for service design, as well as preventive strategies to improve child health and well-being.

